# 

**Published:** 1840-06

**Authors:** 


### WESTERN JOURNAL

OF

### MEDICINE AND SURGERY.

### COLLABORATORS.

EDWARD H. BARTON, M. D. *Professor of the Theory and Practice of Medicine, in the Medical College of Louisiana.*

WILLIAM J. BARBEE, M. D. *Georgetown, Ky.*

GEORGE W. BAYLESS, M. D. *Dissector for the Pathological Cabinet of the Louisville Medical Institute.*

A. H. BUCHANAN, M. D. *Columbia, Tennessee.*

CHARLES CALDWELL, M. D. *Professor of the Institutes of Medicine and Medical Jurisprudence, in the Louisville Medical Institute.*

SAMUEL A. CARTWRIGHT, M. D. *Mississippi.*

A. CLAPP, M. D. *New Albany, Indiana.*

JEDEDIAH COBB, M. D. *Professor of Anatomy, in the Louisville Medical Institute.*

JOHN ESTEN COOKE, M. D. *Professor of the Theory and Practice of Medicine, in the Louisville Medical Institute.*

SAMUEL D. GROSS, M. D. *Professor of Surgery in the Louisville Medical Institute.*

JOHN HARDIN, M. D. *Munfordsville, Ky.*

JOHN P. HARRISON, M. D. *late Professor of Materia Medica, in the Cincinnati College.*

JOHN F. HENRY, M. D. *Bloomington, Illinois.*

SAMUEL P. HILDRETH, M. D. *Marietta, Ohio.*

SAMUEL HOGG, M. D. *Nashville, Tennessee.*

M. Z. KREIDER, M. D., *Lancaster, Ohio.*

MOSES L. LINTON, M. D. *Springfield, Kentucky.*

HENRY MILLER, M. D. *Professor of Obstetrics and the Diseases of Women and Children, in the Louisville Medical Institute.*

JOHN W. MONETTE, M. D. *Washington, Mississsippi.*

HENRY PERRINE, M. D. *Florida.*

SAMUEL B. RICHARDSON, M. D. *Louisville, Ky.*

JOHN L. RIDDELL, M. D. *Professor of Chemistry in the Medical College of Louisiana.*

LANDON C. RIVES, M. D. *late Professor of Obstetrics, in the Medical Department of the Cincinnati College.*

CHARLES W. SHORT, M. D. *Professor of Materia Medica, in the Louisville Medical Institute.*

G. TROOST, M. D. *Professor of Chemistry and Mineralogy, in the University of Nashville.*

AMASA L. TROWBRIDGE, M. D. *Professor of Surgery, Willoughby University, Ohio.*

JOHN A. WARDER, M. D. *Cincinnati, Ohio.*

WILLIAM WOOD, M. D. *Cincinnati, Ohio.*

### INDEX.

A

Abernethy as a Lecturer, 471.
Abscess of the Kidney, 25
Acid-Nitro-Muriatic as a remedial agent, 317.
Aconitine—Cartwright on 473.
Adhesive application, 352.
Affections of the brain v. s. in 68.
Alabama—fevers in 482.
Algiers—state of medicine in 472.
Analysis of an article on hanging, 264.
Analysis of Paroquet Springs, 395.
Anasarca—case of 368.
Anatomy, Pathological—Gross, 85.
Anatomy, Surgical, 484.
Animal magnetism, 388.
Anus, artificial cure of 481.
Army, candidates for admission into 384.
Arsenic, iron its antidote, 61.
Arsenic, poisoning with 328.
Asylums for Deaf and Dumb, 373.
Asylums, Lunatic, 380.

B

Baltimore College of Dental Surgery, 369.
Barbee on Milk Sickness, 178.
Bayless on Tubercle, 196.
Becks statistics of medical colleges of U. S. 273.
Beheaded, remarks on a person who had been 423.
Bigelow on Puerperal Convulsions, 229.
Bigelow on Tænia Solium, 229.
Bowling on Dysenteric Fever, 166.
Boston Med. Journal, 484.
Brain, v. s. in affections of 68.
Brain, concussion of 455.
Brodie, Sir B. on calculus 335.
Broussais, M. history of the last illness of 68.
Burtt on concussion of the Brain, 455.

C

Calculus, Sir B. Brodie on 335.
Cartwright on Jussieua Grandiflora, 428.
Cartwright on new vegetable alkalies, 473.
Child, case of a, with two heads 336.
Child, death from salivation in a, 361.
Children, five at one birth, 60.
Children, convulsions in, 209.
Climate of Tropical Florida, 82.
Cogley on blindness in Pneumonitis, 215.
Combe's phrenological lectures, 379.
Concussion of the brain, 455.
Congenital deafness, cure of, 372.
Consumptives, winter retreat for, 370,
Convulsions in Children, 209.
Creosote in deafness, 470.
Curvatures of the Spine, 59.

D

Davy on the torpedo, 457.

Dawson on Intussusceptio, 193.
Deaf and Dumb, asylums for, 373.
Deafness, cure of congenital, 372.
Deafness cured by Creosote, 470.
Delay, our, 359.
Delphinia in rheumatism, 473.
Dennis' translation of Malgaigne, 293.
Department Editorial, 360.
Dickinson on fracture of skull, 424.
Dysenteric Fever, 166.
Dysmenorrhœa, pessary in, 482.

E

Egypt, medical instruction in, 375.
Electriciiy, Hare on, 355.
Electricity of the Torpedo, 457.
Enterprize, our, 79.
Espy's Lectures on meteorology, 377.
Esquirol on Insanity, 350.
Essay on the fevers of Mississippi, 87.
Excisions, a Monograph on, 293.
Eye, surgical and pathological observations on, 130.

F

Fever, Acetate of Lead in, 218.
Fever, Dysenteric Bilious, 166.
Fever in Alabama, 482.
Fevers of Natchez, Hogg on, 401.
Florida, tropical climate of, 82.
Fractures of the skull, 424.

G

Galvanic Obstetric Forceps, 61.
Girl, Stone in a, relieved by Lithontritie, 339.
Graduates of Medicine in the U. S. 487.
Gross', Pathological Anatomy, 85.
——— proposed work on Surgical Anatomy, 484.

H

Hail-Storm, 378.
Hare on Electricity, 355.
Harris on Fevers in Alabama, 482.
Harrodsburg Springs, 379.
Hemorrhage, internal Uterine, 341.
Hernia cured by fracture of the thigh, 481.
Herpes, caused by Tænia Solium, 229.
Higgason on Secale Cornutum, 29.
Hogg on the fevers of Natchez, 401.
Hosacks Lectures, 33 & 233.
Hospitals of London, Paris, Dublin and Edinburg, 388.
Hospitals of the west, 362.
Hydrated Peroxyde of Iron as an Antidote to Arsenic, 61.

I

Illinois, production of Castor Oil in, 364.
Infants, mortality of, 3.
Ingalls on Scarlatina, 55.
Insanity, Esquirol on, 350.
Institute, Louisville Medical, 86.
Internal Uterine Hemorrhage, 341.
Intussusceptio, 67.
Invalids, subterranean retreat for, 379.
Iron Hydrated Peroxyde of as an Antidote to Arsenic, 61.

J

Journals Medical, origin of, 71.
Jussieua Grandiflora hygenic, 428.

K

Kentucky, Convention of the N. E. Physicians of, 365.
Kidney, abscess of, 25.
Kimball on acetate of lead in fever, 218.

L

Laryngitis, Trosseau and Bellog on, 85.
Lateral curvatures of the spine, 59.
Lightning rods, mode of constructing, 355.
Linton on European Hospitals, 388.
Lithontrite, a case of, in a girl, 339.
Louisville Medical Institute, 86.
Louisiana, South of healthy, 428.
Lunatic Asylums, 380.

M

Magnetism animal exploded, 383.

Malaria, 360.
Malformation, singular case of, 60.
Malgaigne on Excisions, 293.
McDowell on the Eye, 130.
Mechanism versus vitalism, 354.
Medical Colleges of the U. S. 273.
Medical Graduates of the U. S. 477.
Medical Examiner, 256.
Medical Library, 260.
Medical Journals, origin of, 71.
Medicine, Egyptian schools of, 375.
Medical Society of Stark county O. 371.
Medical Society of Tennessee, 397.
Medicine, state of, in Algiers, 472.
Meigs' Midwifery, 280.
Mercurial fumigation, death from, 84.
Meteorology, Espy's Lectures on, 377.
Midwifery, Philadelphia practice of, 280.
Miller on the mortality of Infants, 3.
Millikin on convulsions in children, 209.
Milk-Sickness, 84, 178, 191, 231.
Mississippi, Fevers of, 87.
Monette on Remittent Fever, 87.
Mortality of Infants, 3.
Mortality in New York in 1839, 339.

N

Natchez, Fevers of, 401.
Neuralgia cured by extirpation of a rib, 77.
Neuralgia cured by extraction of a tooth, 337.
Neuralgia, vegetable alkalies in, 473.
New York, mortality of, 339.
Nitro-Muriatic acid as a remedy, 317.

O

Ohio, Stark County, Medical Society, 371.
Ozæna cured by extraction of a tooth, 337.

P

Paralysis, treatment of, in a London Hospital, 336.
Parrish, Dr. death of, 387.
Paroquet Springs, 395.
Pathological Anatomy, Gross on, 85.
Partridge on Creosote, 478.
Pennock's experiments on the heart, 56.
Phrenology, Combe's Lectures on, 379.
Physicians of N. E. Kentucky, Convention of, 365.
Plants, a Catalogne of, 278.
Pessary in Dysmenorrhœa, 482.
Pneumonitis, deafness and blindness from, 215.
Poisoning with arsenic, detection of, 328.
Pregnancy, extra-uterine, 207.
Pseudocyesis Molaris, ergot in, 29.
Puerperal convulsions, 224.
Purpura Hemorrhagica, a case of, 452.
Powell's case of abscess of the kidney, 25.

R

Rain, variation in the quantity of 360.
Remedy for Spine bifida, 362,
Retreat for Invalids, 370, 379.

S

Scarlatina, treatment of, 55.
Scarlatina Simplex, 85.
Searcy's case of Purpura, 452.
Secale Cornutum, 29.
Sexton on Extra-Uterine Pregnancy, 207.
Skull, fracture of, 424.
Short on Milk-Sickness, 231.
Spina bifida, a remedy for, 363.
Spine, lateral curvatures of, 59.
Springs, Clarendon, 370.
Springs, Harrodsburg, 379.
Springs, Paroquet, 395.
Subterranean retreat for invalids,
Surgery, Dental, 369.

T

Tænia Solium, cause of herpes, 229.
Tennessee, Medical Society of, 397.
Tonsils, enlargement of, 317.
Torpedo, electricity of, 457.
Triplets a case of, 368.
Trosseau and Bellog on Laryngitis,
Tubercles a case of, 196.
Tooth decayed, causing ozæna, 337.

U

Urinary diseases, 64.
Uterus, forcible removal of, 478.

V

Variations in the quantity of rain, 360.
Veratria in rheumatism, and neuralgia, 473.

W

West, Commercial Hospitals in, 362.
Weevil, vesicating properties of, 72.

Y

Yellow Fever in the south, 80, 401.

